# No evidence for a causal link between *Helicobacter pylori* infection and Irritable bowel syndrome: a Mendelian randomization study

**DOI:** 10.3389/fmicb.2023.1268492

**Published:** 2024-02-07

**Authors:** Chenchen Wang, Jing Zhang, Fengli Han, Dong Liu, Yuying Han

**Affiliations:** ^1^Laboratory of Tissue Engineering, Faculty of Life Science, Northwest University, Xi’an, China; ^2^Department of Critical Care Medicine, The 940th Hospital of the Joint Logistics Support Force of People’s Liberation Army (PLA), Lanzhou, Gansu, China; ^3^The First Hospital of Northwestern University, Xi’an, China

**Keywords:** *Helicobacter pylori*, Mendelian randomization, irritable bowel syndrome, causality, genome-wide association studies

## Abstract

**Background:**

Although clinical studies have revealed a potential link between *Helicobacter pylori* (*H. pylori*) infection and irritable bowel syndrome (IBS), the causal relationship between them remains unknown. The objective of this study was to investigate whether *H. pylori* infection is causally associated with IBS.

**Method:**

A two-sample Mendelian randomization (MR) analysis using the inverse variance weighted (IVW), weighted mode, weighted median and MR-Egger methods was performed. We used the publicly available summary statistics data sets of genome-wide association studies (GWAS) for *H. pylori* infection in individuals of European descent (case = 1,058, control = 3,625) as the exposure and a GWAS for non-cancer illness code self-reported: IBS (case = 10,939, control = 451,994) as the outcome.

**Results:**

We selected 10 single nucleotide polymorphisms at genome-wide significance from GWASs on *H. pylori* infection as the instrumental variables. The IVW, weighted mode, weighted median and MR-Egger methods all provided consistent evidence that suggests a lack of causal association between *H. pylori* and IBS. MR-Egger regression revealed that directional pleiotropy was unlikely to be biasing the result (intercept = −1e-04; *P* = 0.831). Cochran’s *Q*-test and the funnel plot indicated no evidence of heterogeneity and asymmetry, indicating no directional pleiotropy.

**Conclusion:**

The results of MR analysis support that *H. pylori* infection may not be causally associated with an increased risk of IBS.

## 1 Introduction

Irritable bowel syndrome (IBS) is a chronic functional disorder characterized by abdominal discomfort and changes in bowel habits (Chey et al., 2015; Sultan and Malhotra, 2017). The Rome Foundation Global Study reported a pooled prevalence rate of 4.1% among 54,127 individuals from 26 countries (Sperber et al., 2021). IBS is known to cause distress, morbidity, and disability, and its impact on individuals’ quality of life and healthcare burden cannot be ignored (Chey et al., 2015; Black and Ford, 2020). Therefore, it is crucial to identify the risk factors of IBS and develop targeted treatments. Recent research has increasingly shown that the gut-brain axis and gut microbiota play roles in the development of IBS, potentially explaining its underlying pathogenesis (Holtmann et al., 2016; Pittayanon et al., 2019).

*Helicobacter pylori* is an important gastroenterological pathogen that is highly prevalent worldwide. Approximately 60% of the world’s population has been infected with *H. pylori* (Hooi et al., 2017). Previous studies have reported the associations between *H. pylori* infection and various intra-gastric and extra-gastric diseases, including peptic ulcers, functional dyspepsia, and cholelithiasis (Zhao et al., 2014; Cen et al., 2018). Some studies have suggested that *H. pylori* infection may contribute to the development of IBS (Yakoob et al., 2012; Liang et al., 2020). Meta-analyses have also indicated an increased risk of IBS associated with *H. pylori* infection and that eradication treatment can improve IBS symptoms (Wang et al., 2023). However, other multicenter retrospective studies and meta-analyses have not found evidence supporting this association (Xiong et al., 2016; Ng et al., 2019; Kim et al., 2020; Wang et al., 2022). In addition, we should consider the limitations of these observational studies, which may be subject to biases such as unmeasured or imprecisely measured confounders and reverse causation (Lee et al., 2013).

Mendelian randomization (MR) is an epidemiological analytical technique that utilizes genetic variants as instrumental variables (IVs) to evaluate whether an observational association between the exposure factors and complex disorders is indicative of a causal relationship (Burgess et al., 2015).

In this study, we first conducted a two-sample MR analysis to investigate the potential causal relationship between *H. pylori* infection and the occurrence of IBS. This analysis was based on genome-wide association study (GWAS) data.

## 2 Materials and methods

### 2.1 Mendelian randomization design

**FIGURE 1:**
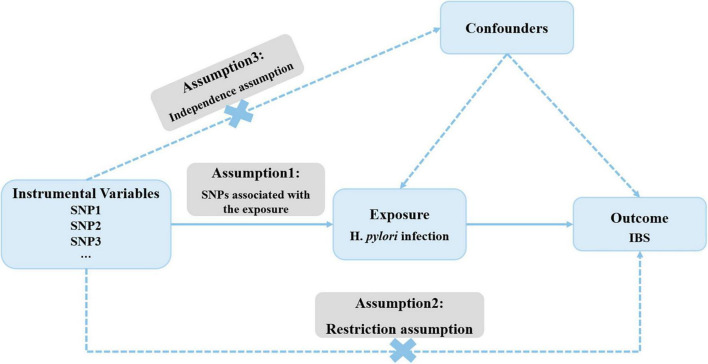
Overall design of Mendelian randomization analyses in the present study.

Figure 1 outlines the framework for the current MR study, which utilized genetic variants as IVs. The validity of MR study rests on three core assumptions: (1) the relevance assumption; (2) the independence assumption; and (3) the exclusion-restriction assumption (Emdin et al., 2017).

### 2.2 Data sources description

The summary data of *H. pylori* infection in GWAS were collected from the publicly available data compiled by the European Bioinformatics Institute (EBI) database. The dataset consisted of 1,058 cases and 3,625 controls of European ancestry. The genetic association of IBS was also generated from the GWAS data sources on EBI, comprising 10,939 European cases and 451,994 European controls. Supplementary Table 1 provides the detailed information of the GWAS data contained in this study.

### 2.3 Selection of genetic instrumental variables for *H. pylori* infection

To obtain the IVs for *H. pylori* infection, the GWAS summary statistics were utilized. A series of quality control measures were implemented to select eligible genetic IVs that fulfilled the assumptions of MR analysis. Firstly, single nucleotide polymorphisms (SNPs) were required to demonstrate genome-wide significance, with a relaxed correlation threshold of *P* < 5 × 10^–6^. Secondly, a linkage disequilibrium (LD) clumping algorithm was used to exclude SNPs in strong LD, with an *R*^2^ < 0.001, a window size of 10,000 kb, and a significance level of *P* < 5 × 10^–8^. Finally, to ensure that the effect alleles were consistent across the exposure and outcome datasets, SNP harmonization was performed, eliminating SNPs with intermediate allele frequencies and ambiguous SNPs with non-concordant alleles. Following these rigorous selection criteria, these SNPs were used as the IVs for subsequent analysis.

### 2.4 Statistical analysis

Two-sample Mendelian randomization was used to estimate the potential causal relationship between *H. pylori* infection and IBS. This analysis was performed using the “TwoSampleMR” packages in R software version 4.0.2. The inverse variance weighted (IVW) method was primarily used for the MR analysis, as it provides a consistent estimates of exposure-outcome associations when the IVs are not pleiotropic (Burgess et al., 2013). Cochran’s *Q* statistics was applied to assess the heterogeneity across individual SNPs (Hemani et al., 2018). Sensitivity analyses were also conducted to verify the robustness of our results. MR-Egger and weighted-median methods were used to explore and adjust for pleiotropy, which refers to the association of genetic variants with more than one variable (Bowden et al., 2015, 2016; Zhu et al., 2016). The threshold for statistical significance was set at a two-sided *P*-value ≤ 0.05.

## 3 Results

### 3.1 Genetic instrumental variable selection

Based on a genome-wide significant threshold of *P* < 5 × 10^–6^, a total of 12 SNPs for *H. pylori* infection were identified. Subsequently, the screening was rigorous as described previously, resulting in the identification of 10 SNPs (rs12591869, rs2169557, rs35030589, rs41263973, rs55871438, rs72708546, rs73512476, rs74045808, rs77516628, rs78825412) (total *R*^2^ of 4.8%) for the associations between *H. pylori* and IBS. All F-statistic values for the obtained instrumental variables (IVs) were greater than 10, indicating no significant weak IV bias. The information on these genetic variants utilized in the MR analyses is detailed in Supplementary Table 2.

### 3.2 Effects of genetically proxied *H. pylori* infection on IBS

**FIGURE 2:**
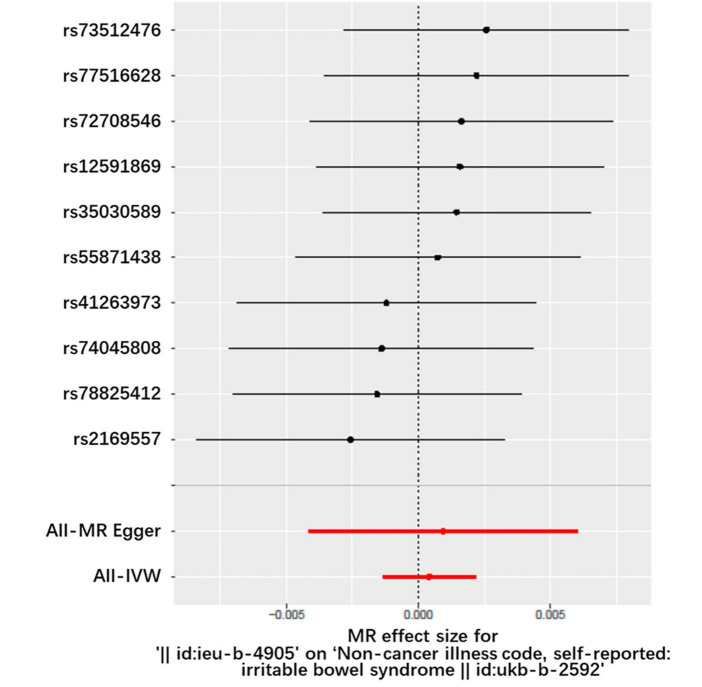
Forest plot of the causal effects of single nucleotide polymorphisms associated with *H. pylori* infection on IBS. The significance of red lines are MR results of MR-Egger test and IVW method.

**FIGURE 3:**
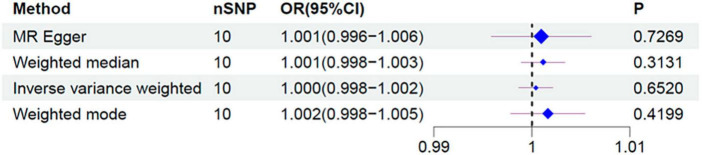
Forest plot for the causal effect of *H. pylori* infection on the risk of IBS. OR, odds ratio; CI, confidence interval.

Figures 2, 3 and Table 1 display the causal estimate of *H. pylori* on IBS. Genetically predicted *H. pylori* infection showed no association with IBS under the IVM (OR = 1.000, 95% CI: 0.998–1.002, *P* = 0.652) and MR-Egger methods (OR = 1.001, 95% CI: 0.996–1.006, *P* = 0.7269). The intercept represents the average pleiotropic effect across the genetic variants. An intercept that differs from zero indicates directional pleiotropy. In this study, MR-Egger regression revealed that directional pleiotropy was unlikely to be biasing the result (intercept = −1e-04; *P* = 0.831). Similar results were obtained when using the weighted median and weighted mode methods (OR = 1.001, 95% CI: 0.998–1.003, *P* = 0.3131 and OR = 1.002, 95% CI: 0.998–1.005, *P* = 0.4199, respectively). Taking all of the above into consideration, the results of the MR analysis may not support a potential causal association between *H. pylori* and IBS.

**TABLE 1:** MR estimates from each method of assessing the causal effect of *H. pylori* on the risk of IBS.

MR method	Number of SNPs	β	Standard error (SE)	OR	95% CI	*P*-value	Cochran *Q* statistic	Heterogeneity *P*-value
MR-Egger	10	0.0009426	0.002606	1.001	0.996–1.006	0.7269	3.658	0.8866
Weighted median	10	0.001142	0.001132	1.001	0.998–1.003	0.3131		
Inverse variance weighted	10	0.0004029	0.0008933	1.000	0.998–1.002	0.652	3.707	0.9296
Weighted mode	10	0.001626	0.001924	1.002	0.998–1.005	0.4199		

### 3.3 Heterogeneity and sensitivity test

**FIGURE 4:**
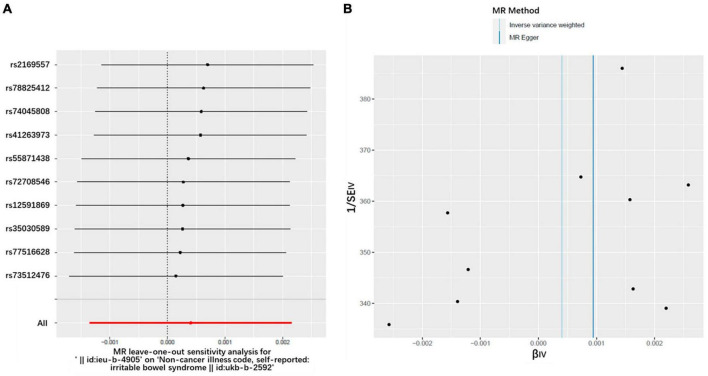
**(A)** MR leave-one-out analysis; **(B)** Funnel plot to assess heterogeneity.

Heterogeneity refers to the variability observed in the causal estimates obtained for each SNP. Low heterogeneity suggests that the results of MR estimates are more reliable. In this study, the Cochran’s *Q*-test revealed no evidence of heterogeneity among the IV estimates based on the individual variants (Table 1). Furthermore, the “leave-one-out” analysis demonstrated that no single SNP was influential in driving the IVW point estimate (Figure 4A). Additionally, the funnel plot displayed no evidence of asymmetry, indicating the absence of directional horizontal pleiotropy (Figure 4B).

## 4 Discussion

In this study, we utilized an MR design to investigate the causal association between *H. pylori* infection and the risk of developing IBS, using publicly available GWAS data. Based on this study, we do not find a significant causal relationship between *H. pylori* infection and IBS.

There is inconsistent evidence regarding the causal relationship between *H. pylori* infection and the risk of IBS. One study conducted on a large cohort showed that *H. pylori* infection was associated with an increased risk of IBS (HR = 4.16; 95% CI: 2.508–6.900), and that eradication therapy can reduce the risk of IBS (Liang et al., 2020; Xiong et al., 2020). A meta-analysis of 31 studies also reached similar results (Wang et al., 2023). However, the relationship between the two has been controversial. Some reports from different countries failed to find the association (Agreus et al., 1995; Locke et al., 2000; Kawamura et al., 2001; Zhao et al., 2010). Additionally, a multicenter retrospective study concluded that *H. pylori* infection may not be related to IBS, and that the symptoms of IBS patients, such as abdominal pain and stool frequency, cannot be improved after *H. pylori* eradication (Xiong et al., 2016). Furthermore, a study found that the prevalence of *H. pylori* infection was lower in the IBS group (Athab, 2017).

The controversy surrounding the relationship between *H. pylori* infection and IBS can be attributed to several reasons. Firstly, as mentioned earlier, observational studies are prone to biases, which may explain the discrepancies (Lee et al., 2013). Secondly, differences in diagnostic approaches could result in unavoidable bias. Some studies used serum or fetal IgG antibodies to diagnose *H. pylori* infection, which may lead to false positive results (Kim et al., 2017; Abdel-Razik et al., 2018; Abo-Amer et al., 2020; Alvarez et al., 2020). As everyone knows, the urea breath test (UBT) has been shown to have the best accuracy among non-invasive tests (Liu et al., 2022). Additionally, subgroup analysis revealed opposite correlations between *H. pylori* infection and IBS when diagnosed using Rome III or non-Rome III criteria (Li et al., 2020). Therefore, it is crucial for the analysis to employ a unified and strict diagnostic criterion. Thirdly, as described in a meta-analysis study (Hooi et al., 2017), the prevalence of *H. pylori* infection and IBS varies across different geographical regions and races, which may introduce selection bias.

Irritable bowel syndrome (IBS) poses a significant medical challenge to society, and the development of a novel treatment for this disease is hindered by the lack of understanding of its etiology and pathogenesis. This study aims to shed light on the pathogenic factors of IBS from the perspective of systems biology. Our MR study has several strengths. Firstly, to the best of our knowledge, this is the first research to reveal the causal relationship between *H. pylori* infection and IBS. Secondly, this study has a large sample size and high statistical efficiency. Thirdly, the results were consistent across all approaches. Additionally, we conducted Cochran’s *Q*-test, MR-Egger intercept test, and various sensitivity analyses, including a leave-one-out analysis and a funnel plot, to test the validity of the conclusion and assess the pleiotropy of IVs. However, this analysis also has several limitations. Firstly, the diagnosis of *H. pylori* infection was based on serum IgG antibodies testing in the datasets. Secondly, the datasets used primarily include the European population, so the results may not be generalizable to other populations. Thirdly, IBS patients were classified based on non-cancer illness codes, which may introduce information bias. Lastly, in order to incorporate a certain number of SNPs, the *P*-value limits were adjusted.

In conclusion, this MR study did not identify a causal effect between *H. pylori* infection and IBS, indicating that IBS patients may not derive any benefits from the prevention or eradication of *H. pylori* infection. However, due to the presence of these limitations, larger prospective studies and additional MR studies may be necessary to evaluate the correlation.

## Data availability statement

The original contributions presented in this study are included in this article/Supplementary material, further inquiries can be directed to the corresponding authors.

## Ethics statement

Ethical approval was not required for the study involving humans in accordance with the local legislation and institutional requirements. Written informed consent to participate in this study was not required from the participants or the participants’ legal guardians/next of kin in accordance with the national legislation and the institutional requirements.

## Author contributions

CW: Data curation, Software, Writing – original draft. JZ: Writing – original draft. FH: Writing – review & editing. DL: Writing – review & editing. YH: Supervision, Writing – review & editing.
