# Supplementary material for: No evidence for a causal link between Helicobacter pylori infection and Irritable bowel syndrome: a Mendelian randomization study
Source: Front Microbiol. 2024 Feb 7;14:1268492. doi: 10.3389/fmicb.2023.1268492 (PMC10879563; doi:10.3389/fmicb.2023.1268492)
Supplement: Supplementary file 1 [file Table_1.DOCX]

Supplementary Material

# Supplementary Table 1

| **Supplementary Table1 Details of the studies included in the Mendelian randomization analyses.** | | | | | | |
| --- | --- | --- | --- | --- | --- | --- |
| **Phenotype** | **Consortium or anthor** | **Ethnicity** | **Sample size** | **Year** | **Number  of SNPs** | **Web source** |
| *H. polyri* infection | EBI | European | 1,058 cases and 3,625 controls | 2021 | 7,247,045 | https://gwas.mrcieu.ac.uk/datasets/ieu-b-4905/ |
| IBS | Ben Elsworth | European | 10,939 cases and 451,994 controls | 2018 | 9,851,867 | https://gwas.mrcieu.ac.uk/datasets/ukb-b-2592/ |
| SNP, single-nucleotide polymorphisms; *H. pylori,* Helicobacter pylori; EBI, European Bioinformatics Institute; IBS, irritable bowel syndrome. | | | | | | |

| **Supplementary Table 2 Instrumental SNPs of *H. pylori* infection and F statistics.** | | | | | | | | |
| --- | --- | --- | --- | --- | --- | --- | --- | --- |
| **Instrumental SNP** | **Effect allele** | **Other allele** | **EAF** | **BETA** | **SE** | ***P*** | ***R2*** | ***F*** |
| rs12591869 | A | C | 0.26981 | -0.128595 | 0.0265143 | 0.0265143 | 0.004997901 | 23.51268996 |
| rs2169557 | T | C | 0.48846 | -0.105911 | 0.0230674 | 0.0230674 | 0.004481363 | 21.07168801 |
| rs35030589 | A | G | 0.13205 | -0.175163 | 0.0342852 | 0.0342852 | 0.005542852 | 26.09070764 |
| rs41263973 | A | G | 0.033534 | 0.315775 | 0.0672338 | 0.0672338 | 0.004688297 | 22.04929232 |
| rs55871438 | C | T | 0.039419 | 0.29919 | 0.0647964 | 0.0647964 | 0.004532059 | 21.31114948 |
| rs72708546 | A | G | 0.059115 | -0.229692 | 0.0481386 | 0.0481386 | 0.004838095 | 22.75722538 |
| rs73512476 | T | G | 0.076735 | 0.212576 | 0.0440791 | 0.0440791 | 0.004941825 | 23.24756698 |
| rs74045808 | T | C | 0.10756 | -0.17464 | 0.038012 | 0.038012 | 0.004487131 | 21.09893273 |
| rs77516628 | T | A | 0.088953 | 0.18787 | 0.0401253 | 0.0401253 | 0.004659356 | 21.91254239 |
| rs78825412 | A | C | 0.032426 | 0.31781 | 0.0685235 | 0.0685235 | 0.004572372 | 21.50158746 |

# Supplementary Table 2
